# Supplementary material for: Cost-effectiveness of the SMILE intervention compared with usual care for people with severe mental illness: A randomized clinical trial
Source: PLoS One. 2025 Jan 6;20(1):e0313100. doi: 10.1371/journal.pone.0313100 (PMC11703068; doi:10.1371/journal.pone.0313100)
Supplement: Attachment — Submitted filename: Answer editorial comments.docx [file pone.0313100.s004.docx]

**Authors’ response on editorial comments**

**Comment 1:**

1. The in-house editorial staff feels that your study meets the World Health Organization definition of a clinical trial because it is a prospective study in which participants were assigned to SMILE intervention to investigate the effects on economic outcomes. Please change your manuscript’s article type to ‘Clinical Trial’ when you resubmit your manuscript.

We’ve changed the title and now refer to the paper as a clinical trial. Please not that this is an economic evaluation alongside a clinical trial, where economic outcomes are being communicated. Detailed information about the effectiveness of the intervention can be found elsewhere:

- Walburg, F. S., van Meijel, B., Hoekstra, T., Kol, J., Pape, L. M., de Joode, J. W., van Tulder, M., & Adriaanse, M. (2023). Effectiveness of a Lifestyle Intervention for People With a Severe Mental Illness in Dutch Outpatient Mental Health Care: A Randomized Clinical Trial. *JAMA psychiatry*, *80*(9), 886–894. <https://doi.org/10.1001/jamapsychiatry.2023.1566>

We refer to this paper frequently throughout the manuscript.

**Comment 2:**

2. PLOS ONE requires that all clinical trials are registered in an appropriate registry (the WHO list of approved registries is at      [https://www.who.int/clinical-trials-registry-platform/network/primary-registries"](https://www.who.int/clinical-trials-registry-platform/network/primary-registries%22) <https://www.who.int/clinical-trials-registry-platform/network/primary-registries> and more information on trial registration is at <http://www.icmje.org/about-icmje/faqs/clinical-trials-registration/)>. Please state the name of the registry and the registration number (e.g. ISRCTN or ClinicalTrials.gov) in the submission data and on the title page of your manuscript.

a) Please provide the complete date range for participant recruitment and follow-up in the methods section of your manuscript.

This already was added to the manuscript in the previous version.

b) If you have not yet registered your trial in an appropriate registry, we now require you to do so and will need confirmation of the trial registry number before we can pass your paper to the next stage of review. Please include in the Methods section of your paper your reasons for not registering this study before enrolment of participants started. Please confirm that all related trials are registered by stating: “The authors confirm that all ongoing and related trials for this drug/intervention are registered”. Please see <http://journals.plos.org/plosone/s/submission-guidelines#loc-clinical-trials> for our policies on clinical trials.

This has been added to the manuscript and additional information regarding registration as well.

**Comment 3:**

3. Please note that you must upload a completed CONSORT flowchart as figure 1 of your manuscript. Blank copies of this document and information regarding CONSORT can be found via the following link: (<https://www.equator-network.org/reporting-guidelines/consort/>).

We have added a filled in CONSORT checklist. However, we believe that this is not the best checklist to assess the reporting quality of our manuscript. Since we’re performing an economic evaluations, the CHEERS checklist is more appropriate (<https://www.equator-network.org/reporting-guidelines/cheers/>). We’ve also included a filled in version of this checklist as a supplementary file. Regarding the CONSORT flowchart, this is already included in the effectiveness paper that we refer to in our manuscript. Including this flowchart would complicate describing the results because in the economic evaluation we’ve used multiple imputation to deal with missing data. This means that all observations for the whole follow-up period have been used in the analysis rather than only the observations of patients that have completed the trial. This does not line up with the CONSORT flowchart in Walburg et al. (2023) (<https://doi.org/10.1001/jamapsychiatry.2023.1566>)

**Comment 4:**

4. Please note that you must upload a completed CONSORT checklist as a supporting information file. Blank copies of this document and information regarding CONSORT can be found via the following link: (<https://www.equator-network.org/reporting-guidelines/consort/>). If your clinical trial uses a non-randomized design, you may wish to submit a TREND checklist (<http://www.cdc.gov/trendstatement>), in place of the CONSORT checklist.

See answer comment 3.

**Comment 5:**

5. Please upload a copy of your trial study protocol as a supporting information file. By the study protocol, we mean the complete and detailed plan for the conduct and analysis of the trial that the ethics committee approved before the trial began. Please send this in the original language. If this is in a language other than English, please also provide a translation. Please detail any deviations from this study protocol in the Methods section of your manuscript. Your study protocol will be made available to the editors and reviewers, and will be published as supporting information with your manuscript if accepted for publication. (If you do not agree to this, we will not be able to publish your manuscript). If you have formally published a study protocol for your trial in a journal then you should cite this in your manuscript, but you still need to send us the original document

We’ve included the trial protocol as well as the published design paper:

- Walburg, F. S., van Meijel, B., van Tulder, M. W., & Adriaanse, M. C. (2019). Cost-effectiveness of a lifestyle intervention for people with a serious mental illness (SMILE): design of a pragmatic cluster-randomised controlled trial. *BMC psychiatry*, *19*(1), 151. https://doi.org/10.1186/s12888-019-2132-5
