# Supplementary material for: Cost-effectiveness of the SMILE intervention compared with usual care for people with severe mental illness: A randomized clinical trial
Source: PLoS One. 2025 Jan 6;20(1):e0313100. doi: 10.1371/journal.pone.0313100 (PMC11703068; doi:10.1371/journal.pone.0313100)
Supplement: Attachment — Submitted filename: 19122023_KEA SMILE_comment.docx [file pone.0313100.s005.docx]

**Cost-effectiveness of the SMILE intervention compared with usual care for people with severe mental illness: A Randomized Clinical Trial**

**Short title: Cost-effectiveness of the SMILE intervention**

**Mohamed El Alili^1,2*^, Berno van Meijel^3,4,5^, Maurits W. van Tulder^6^ and Marcel Adriaanse^1^**

Affiliations:

^1^ Department of Health Sciences, Vrije Universiteit Amsterdam, Faculty of Science, Amsterdam Public Health Research Institute, Amsterdam, The Netherlands

^2^ Zorginstituut Nederland, Diemen, The Netherlands.

^3^ Inholland University of Applied Sciences, Department of Health, Sports & Welfare, Research Group Mental Health Nursing, Amsterdam, The Netherlands

^4^ Amsterdam UMC (VUmc), Department of Psychiatry, Amsterdam Public Health research institute, Amsterdam, The Netherlands

^5^ Parnassia Psychiatric Institute, Parnassia Academy, The Hague, The Netherlands

^6^ Department Clinical, Neuro- & Developmental Psychology, Faculty Behavioural and Movement Sciences, Vrije Universiteit Amsterdam, Amsterdam, The Netherlands

* Corresponding author

E-mail: [m.elalili@vu.nl](mailto:m.elalili@vu.nl)

Word count: 3158 (no maximum)

Tables: 2

Figures: 1

# Abstract

**Objectives:** Only studying effectiveness of lifestyle interventions for people with severe mental illness (SMI) is insufficient for policy making. As budgets for healthcare are limited, policy makers face the problem of allocating scarce healthcare resources. Cost-effectiveness studies are needed, but currently cost-effectiveness studies of lifestyle interventions for people with SMI delivered in ambulatory care are limited. The aim of this current study was to evaluate the cost-effectiveness of a lifestyle intervention for people with SMI living in the Dutch community in comparison with usual care.

**Methods and findings:** An economic evaluation was performed using a societal perspective alongside the Severe Mental Illness Lifestyle Evaluation (SMILE) pragmatic cluster randomized controlled trial. The SMILE lifestyle intervention is a one-year, group-based intervention delivered by trained mental healthcare workers. Costs, body weight change and quality of life were assessed at baseline, 6 and 12 months. Mixed models were used to estimate incremental costs and effects between the treatment group and the usual care group. Overall, the SMILE intervention resulted in lower total costs compared to the usual care group (-€719, 95% CI -7133 ; 3897). The effect difference between the intervention and usual care groups was -3.76 (95% CI -6.30 ; -1.23) kilograms for body weight and -0.037 (95% CI -0.083 ; 0.010) for QALYs.

**Conclusions:** Overall, the SMILE intervention resulted in lower total costs compared to the usual care group and was cost-effective for body weight change. However, the SMILE intervention does not seem cost-effective with regards to QALYs. More cost-effectiveness studies in other countries and other settings are needed to gain further insight into the cost-effectiveness of lifestyle interventions for people with SMI.

**Trial registration & funding**

The protocol was approved by the medical ethics committee of the VU University Medical Centre (NL60315.029.17, registration number 2017.418), registered in the Netherlands Trial Register (Identifier: NTR6837, <https://trialsearch.who.int/Trial2.aspx?TrialID=NTR6837>). The data as registered in the Netherlands Trial Register are automatically included in the International Clinical Trial Registry Platform of the World Health Organization (WHO). The authors confirm that all ongoing and related trials for this intervention are registered. This study was funded by grant 80–84300–98-72012 from the Netherlands Organization for Health Research and Development (ZonMW). The funder had no role in the design and conduct of the study; collection, management, analysis, and interpretation of the data; preparation, review, or approval of the manuscript; and decision to submit the manuscript for publication.

**Conflict of interest**

The authors declare no conflict of interest.

**Author summary**

**Why was this study done?**

- People with severe mental illness (SMI) have a life expectancy 15 to 20 years lower than the general population, primarily attributable to cardiometabolic disorders.
- Lifestyle interventions for people with SMI can improve health and reduce cardiometabolic risk, but lifestyle is not yet integrated in routine mental health care for this population.
- Few studies have evaluated the effectiveness of lifestyle interventions in this population, but little is known about their cost-effectiveness.

**What did the researchers do and find?**

- The study was performed in the Netherlands in 21 Flexible Assertive Community Treatment (FACT) teams delivering care for people with SMI.
- We conducted an economic evaluation alongside a randomized controlled trial to assess the cost-effectiveness of a lifestyle intervention for people with a SMI living in the community in comparison with usual care.
- We used a societal perspective, including all relevant cost of the interventions, healthcare utilization, patient and family related cost, and cost of productivity losses.
- 126 clients participated in the intervention group and 97 in the control group.
- The lifestyle intervention resulted in lower total costs and more body weight loss (-3.8 kilograms (95% CI -6.3 ; -1.2) compared with the control group. Quality adjusted life years (QALYs) were lower in the lifestyle intervention group (-0.037 (95% CI -0.083 ; 0.010)), though not statistically significant.

**What do these findings mean?**

- Our findings suggest that the lifestyle intervention is cost-effective for body weight loss, but not for QALYs.
- Implementation of the lifestyle intervention in routine mental health care for people with SMI might be opportune if weight loss is considered the most important outcome.

# Introduction

People with severe mental illness (SMI) have a lower life expectancy of 15 to 20 years compared to the general population [1-3]. Though the number of unnatural deaths due to suicide and accidents in patients with schizophrenia or bipolar disorder is high[4, 5], physical conditions account for approximately 70% of deaths [4, 6]. Approximately 33% of patients with bipolar disorder and 25% of patients with schizophrenia die from cardiovascular disease [7-9]. Risk factors that contribute to cardiovascular disease such as unhealthy nutrition patterns, low physical activity, smoking, genetic vulnerability, and low access to healthcare are common within this patient population [1, 10-13]. On top of that, antipsychotic drugs, which are frequently part of treatment, are associated with an increased risk of gaining weight, dyslipidaemia and diabetes mellitus [12]. However, people with SMI receive fewer interventions and benefit less from treatment of physical diseases than the general population [1, 5].

In The Netherlands, outpatient mental health care for people with SMI living in the community is commonly offered by Flexible Assertive Community Treatment (FACT) teams. Routine care within FACT-teams consists of multidisciplinary treatment and care by individual case management in the community. Care is determined by the needs of the patient; unstable patients with a high risk of relapse receive intensive assertive outreach care [14]. Awareness of the importance of integrating physical health care within routine mental health care for this population is rising. An increasing number of clinical guidelines for mental health care for people with SMI include a focus on physical health [13]. Although many studies have been conducted [13, 15], evidence for long-term sustainability of lifestyle interventions for people with SMI is still limited [16-19], and implementation into routine care is lacking [19, 20]. Also, little is known regarding the cost-effectiveness of lifestyle interventions within this population as studies often do not describe cost data or address cost-effectiveness [13, 17, 21, 22]. Studying the effectiveness of lifestyle interventions alone is insufficient for policy making. As budgets for healthcare are limited, policy makers face the problem of allocating scarce healthcare resources. Adding lifestyle interventions to routine care may increase costs, such as costs of training staff and extra hours spend on intervention sessions. It is important to know whether these additional costs are worth the potential added benefits. By performing economic evaluations of lifestyle interventions, we gain insight in the cost-effectiveness of these interventions and assist policy makers in establishing priorities within the cost-constrained healthcare budgets. Hence, the aim of this current study was to evaluate the cost-effectiveness of a lifestyle intervention for people with SMI living in the Dutch community in comparison with usual care.

# Methods

## Study design

An economic evaluation from a societal perspective was performed alongside a pragmatic cluster randomized controlled trial (the SMILE-study). Follow-up duration was one year. The design of the SMILE-study was described elsewhere [23], as well as the process evaluation [24] , experiences of people with SMI and health care professionals with the SMILE intervention [25] and the effectiveness of the SMILE intervention [26]. The protocol was approved by the medical ethics committee of the VU University Medical Centre (NL60315.029.17, registration number 2017.418), registered in the Netherlands Trial Register (Identifier: NTR6837, <https://trialsearch.who.int/Trial2.aspx?TrialID=NTR6837>). The data as registered in the Netherlands Trial Register are automatically included in the International Clinical Trial Registry Platform of the World Health Organization (WHO). The authors confirm that all ongoing and related trials for this intervention are registered. Measurements took place at baseline, six and 12 months. Weight was also measured at three months. Questionnaires were completed on hard copy to make the questionnaires more accessible for participants. All participants received 10 euros for each of the measurements performed at baseline, six and 12 months.

## Setting and participants

The study was conducted within 21 FACT-teams from eight mental healthcare centres in the Netherlands. The recruitment for the trial was conducted from the 1^st^ of January 2018 to the 1^st^ of January 2019 with a follow-up of one year. Participants were adults (18 years or older), had a BMI of 27 or higher, and received care from one of the participating FACT-teams. All participants provided written consent. Exclusion of participants was based on cognitive impairments that could interfere with actively participating in the intervention, contra-indications such as an acute psychiatric crisis (assessed by the FACT-teams’ healthcare professionals), inability to communicate in the Dutch language, or (planning for) pregnancy or breastfeeding.

## Randomization

We performed cluster randomization at the level of FACT-teams. One centre participated with only an intervention team, as their control team dropped out of the study after randomisation. A statistician without further involvement in the study performed the randomization using a computerized random number function in Microsoft Excel.

## Intervention

The SMILE intervention is a group-based intervention which focusses on lifestyle changes with an emphasis on a healthy diet and physical activity. In total, there were 30 group sessions throughout the year. People with SMI followed group sessions weekly during the first six months and monthly during the last six months. Group sessions were given by trained FACT-team healthcare professionals. The intervention was primarily modelled after the STRIDE intervention [27-29]. A more detailed description of the SMILE intervention can be found elsewhere [23-27].

## Economic evaluation

Both a cost-effectiveness analysis (CEA) and cost-utility analysis (CUA) were performed with body weight change (in kilograms) and quality adjusted life years (QALYs) as primary outcomes, respectively. For QALYs, the EuroQol-5D-5L (EQ-5D-5L) questionnaire was used [30]. We converted utility scores using the Dutch EQ-5D tariff with a range from –0.446 to 1, where negative utilities indicate a health state that is valued as worse than death, and 1 indicates full health [31]. The EQ-5D-5L contains five dimensions (mobility, self-care, usual activities, pain/discomfort and anxiety/depression) with five response levels (no problems, slight problems, moderate problems, severe problems or extreme problems) [32].

## Costs

Costs were measured from a societal perspective. Costs were converted to 2020, if necessary, using Dutch consumer price indexes [33]. The time horizon of the economic evaluation was 1 year, which means that discounting is not necessary.

#### Intervention costs

We calculated the costs of the intervention using a bottom-up approach. Intervention costs consisted of all costs that were associated with the execution of the intervention. This included the intervention materials, costs of group leaders per hour multiplied by the hours spend on their preparation per group session and the time of the group sessions themselves, time spent on training for group leaders, and travel costs to each group session.

#### Healthcare utilization costs and productivity costs

To reduce the burden of participating in the study, we used an adapted and shorter questionnaire (based on the TIC-P questionnaire) for measuring healthcare utilization and productivity costs [34]. In this questionnaire, we collected data regarding quantity of healthcare that participants received, such as contacts with FACT-team staff (i.e., nurses and psychologists), physiotherapists, general practitioners, outpatient visits, and informal care from relatives or friends. Dutch standard cost prices were used when available [35]. For prices that were not available at least five sources were consulted (e.g., job sites) and an average was calculated. For productivity loss, costs of sick leave were calculated using the mean wage per hour per gender and 5-years age group. Presenteeism costs were calculated by multiplying hours with the mean wage per hour per gender and age group.

In addition, medication use was collected based on the most recent pharmacy overview lists of participants. All medication was grouped into respective medication groups based on the Anatomical Therapeutic Chemical (ATC) code [https://www.whocc.no/]. To calculate medication costs per group we used the highest Dutch costs of each medication based on prices reported by the Dutch National Health Care Institute (Zorginstituut Nederland; [www.medicijnkosten.nl](http://www.medicijnkosten.nl)). We calculated average medication costs by multiplying the daily dose and the average costs of the respective medication group.

## Statistical analysis

The cost-effectiveness analyses were conducted according to the intention-to-treat principle. Missing data were imputed using Multiple Imputation with Chained Equations (MICE) [36]. Cost and effect data were assumed to be missing at random, which means that missing observations are explained by observed variables [37]. The imputation model included outcome variables and predictor variables that either differed at baseline, were related to missing data, or were associated with the outcome (see Table 1 for variables included in the imputation model). To account for the skewed distribution of cost data, predictive mean matching was used in MICE [38]. The number of imputed datasets was increased until the loss of efficiency was less than 5%, resulting in five imputed datasets [38]. Each of the imputed datasets was analysed separately as described below. Results from the multiple datasets were pooled using Rubin’s rules [39].

Mixed models were used to estimate incremental costs and effects between the treatment group and usual care group. This means that we corrected for clustering in the data by allowing the intercepts to vary between clusters (i.e., random intercepts model). For costs and QALYs, a two-level structure was used where FACT-teams and patients represented the first and second level, respectively. For the difference in body weight, an additional level accounted for repeated observations within patients (i.e., patients’ body weight measures at different time points) [40, 41]. Hence, a three-level structure was used where these repeated patients’ observations were nested within patients, which were nested within FACT-teams. This allowed for estimation of an overall effect over time [42]. QALYs were adjusted for baseline utilities [43]. Weight was additionally adjusted for confounders (see Table 1 for list of confounders). Incremental Cost-Effectiveness Ratios (ICERs) were calculated by dividing the incremental costs by incremental effects. Bias-corrected bootstrapping was used to estimate statistical uncertainty (5000 replications). Statistical uncertainty surrounding ICERs was illustrated by plotting the bootstrapped cost-effect pairs on a cost-effectiveness plane (CE-plane). Cost-effectiveness acceptability curves (CEACs) were estimated, which demonstrate the probability that the intervention is cost-effective compared to usual care for a range of different ceiling ratios (i.e., the willingness-to-pay threshold for one point effect extra) [44]. CEACs were estimated using the parametric p-value approach for incremental net-monetary benefits (INMBs) [45]. In the Netherlands, the generally used willingness-to-pay threshold for healthcare ranges between 20,000 and 80,000 Euros per QALY gained depending on the burden of disease [46]. For outcome measures such as weight, no formal willingness-to-pay threshold has been determined. Analyses were performed in StataSE 16^®^ (StataCorp LP, CollegeStation, TX, US).

To check the robustness of the results, two sensitivity analyses were performed. The economic evaluation was performed without adjustment for confounders (SA1) and an analysis was performed including an outlier (SA2). The outlier concerned a patient that received 140 hours of home care in 24 weeks, which had a large impact on the estimation of home care costs.

# Results

**Patient flow and sample characteristics**

The study population included 11 lifestyle intervention FACT-teams (126 participants) and 10 usual care FACT-teams (98 participants). In the main analysis of the economic evaluation, one outlier was removed from the usual care group. This was due to a patient that received 20 hours of home care per day for a consecutive 24 weeks. Further details about patient flow and baseline characteristics are reported elsewhere (discrepancies in the number of patients analysed between the economic evaluation and the effectiveness study are due to the use of multiple imputation) [26].

## Costs

For most cost categories, costs were lower in the intervention group than in the usual care group, but not statically significant (see Table 1). When costs were higher in the intervention group compared to the usual care group, these were significantly higher, except for absenteeism costs as estimated by the human capital approach. The largest difference in costs between the intervention and the usual care group was found for home care (i.e., -€1724), while the smallest difference in costs between intervention and usual care groups was found in loss of unpaid work (i.e., -€6). Total societal costs were €690 lower (95% CI -6348 ; 3386) in the intervention group compared to the usual care group; this difference was not statistically significant.

## Cost-effectiveness

The difference in *body* *weight change* (in kilograms) between the intervention group and the usual care group was -3.76 kilograms, indicating that over time the intervention resulted in a weight loss of -3.76 kilograms (95% CI -6.30 ; -1.23) more than usual care (see Table 2). This difference was statistically significant. For body weight, the intervention was dominant compared to the control group, since effects were larger and costs were lower. The CE-plane (Figure 1a) shows that most of the bootstrapped cost-effect pairs are situated in the south-east quadrant of the plane confirming more weight loss (i.e., weight loss is a favourable outcome, hence a loss of weight is inverted on the CE-plane) and lower costs in the intervention group as compared to the usual care group. The CEA curve (Figure 1b) shows that the probability that the intervention is cost-effective in comparison with usual care is 0.59, 0.92 and 1.00 at willingness-to-pay values of €0, €1000 and €10,000 per kilogram body weight loss, respectively.

The difference in *QALYs* between the intervention and usual care group was -0.037 (95% CI -0.083 ; 0.010), which was not statistically significant. The ICER for QALYs was 19481, indicating that €19481 is saved in the intervention group in comparison with the usual care group when 1 QALY is lost. The CE-plane (Figure 1c) shows that the bootstrapped cost-effect pairs are mainly located in the south-west quadrant confirming the smaller effects and lower costs in the intervention group as compared to the usual care group. The CEA curve (Figure 1d) shows that the probability that the intervention is cost-effective in comparison with usual care is 0.59, 0.50 and 0.38 at willingness-to-pay values of €0, €20,000 and €50,000 per QALY gained, respectively.

## Sensitivity analyses

Performing the cost-effectiveness analysis without adjustment for confounders (SA1) gives relatively similar results (Table 2). However, the QALY difference increased from -0.037 to -0.081, which impacted the ICER (€8867 per QALY lost instead of €19481 per QALY lost). Including the outlier (SA2) had a much larger impact on the cost-effectiveness results. Although the QALY difference remained relatively similar (-0.037 to -0.033), the cost saving increased from €719 to €2993. This was due to a patient in the usual care group that received 20 hours of home care per day for a consecutive 24 weeks. Consequently, it notably changed the ICERs (€89376 per QALY lost instead of €19481 per QALY lost) and the distribution of the bootstrapped cost-effect pairs on the CE-plane.

**Table 1.** Multiply imputed costs per patient and overall effects over time

|  | | Intervention group  (N = 126) | Usual care group  (N = 97) | Main analysis  (N = 223) | Including outlier*  (N = 224) |
| --- | --- | --- | --- | --- | --- |
| **Outcomes** | | **Mean (SE)** | | **Mean difference (95%CI)^§^** | **Mean difference (95%CI)^§^** |
| Body weight (kg) | T0 (baseline)  T1 (3 months)  T2 (6 months)  T3 (12 months) | 101.28 (1.75)  99.22 (1.87)  97.97 (1.89)  97.66 (2.09) | 102.39 (1.83)  102.12 (1.91)  102.17 (1.82)  102.52 (2.04) | -3.76 (-6.77 ; -0.75) ^#, †^ | -3.66 (-6.66 ; -0.66)^#, †^ |
| QALY | | 0.64 (0.020) | 0.73 (0.025) | -0.037 (-0.085 ; 0.012) ^†^ | -0.033 (-0.081 ; 0.014) ^†^ |
| **Healthcare costs** | |  |  |  |  |
| Primary care  Secondary care  Health contacts  Health contacts within FACT team  Admission  Home care  Informal care  Medication  Intervention | | 255 (59)  507 (60)  406 (71)  1881 (150)  1340 (602)  1026 (207)  1076 (219)  1630 (182)  930 (-) | 138 (18)  245 (43)  486 (143)  1927 (221)  2436 (1100)  2750 (2006)  465 (142)  1794 (190)  0 (-) | 117 (41 ; 216)  262 (122 ; 399)  -80 (-413 ; 177)  -46 (-575 ; 417)  -1096 (-3772 ; 844)  -1724 (-6519 ; 504)  611 (165 ; 1117)  -164 (-499 ; 158)  930 (-) | 118 (41 ; 216)  264 (124 ; 403)  -102 (-443 ; 154)  -44 (-578 ; 409)  -1071 (-3761 ; 849)  -3601 (-10276 ; 212)  616 (164 ; 1114)  -157 (-495 ; 161)  930 (-) |
| **Lost productivity costs** | |  |  |  |  |
| Absenteeism FCM  Absenteeism HCA  Presenteeism  Loss of unpaid work | | 1173 (363)  1425 (474)  545 (312)  100 (20) | 1190 (417)  1408 (536)  23 (18)  106 (32) | -17 (-1083 ; 986)  17 (-1330 ; 1305)  522 (155 ; 1182)  -6 (-69 ; 50) | -4 (-1085 ; 976)  31 (-1309 ; 1326)  522 (149 ; 1178)  -5 (-67 ; 49) |
| **Total societal costs FCM** | | 10870 (1117) | 11559 (2583) | -690 (-6348 ; 3386) | -2534 (-9724 ;2486) |
| **Total societal costs HCA** | | 11122 (1159) | 11778 (2619) | -656 (-6350 ; 3561) | -2498 (-9550 ;2662) |
| *One case was added in the control group, bringing the total sample size to n = 224. This concerned the outlier, which was due to a patient in this group that received 20 hours of home care per day for a consecutive 24 weeks.  ^§^Uncertainty around cost differences estimated using the non-parametric bootstrap with 5000 replications (bias-corrected intervals). The presented cost differences are unadjusted.  ^#^Overall effect over time (12 months).  ^†^Covariates included in the mixed model for weight were treatment indicator, baseline weight, gender, primary diagnosis of mental disorder, having a partner and smoking. The mixed model for QALYs was adjusted for baseline utility.  Multiple imputation model consisted of variables that differed at baseline, were related to missing data or were associated with the outcome: gender, diagnosis of mental disorder, smoking at baseline, smoking at 12 months, baseline costs of health contacts outside FACT team, age, weight at baseline, body mass index at baseline, education, home care costs at baseline, utility at baseline, heart rhythm disorder and diabetes. The imputation procedure was stratified for treatment arm and the cluster indicator variable was added to the imputation model to adjust for clustering in the imputation procedure.  FACT = flexible assertive community treatment, FCM = friction cost method, HCA = human capital approach, QALY = Quality adjusted life-year, SE = standard error, 95%CI = 95% confidence interval. | | | | | |

**Table 2.** Results of the cost-effectiveness analyses

| **Outcome** | **Mean cost difference (95% CI)*** | **Mean effect difference (95% CI)** | **ICER** | **Distribution of the Cost-effectiveness plane (%)** | | | |
| --- | --- | --- | --- | --- | --- | --- | --- |
| **Main analysis** |  |  |  | **NE** | **SE** | **SW** | **NW** |
| Body weight | -719 (-7133 ; 3897)* | -3.76 (-6.30 ; -1.23) ***^,^**^†^ | 191 | 39% | 61% | 0% | 0% |
| QALYs | -719 (-7133 ; 3897)* | -0.037 (-0.083 ; 0.010) ***^,^**^†^ | 19481 | 1% | 4% | 57% | 38% |
| **Unadjusted analysis (SA1)** |  |  |  |  |  |  |  |
| Body weight | -719 (-7133 ; 3897)* | -4.16 (-8.17 ; -0.16)* | 173 | 38% | 60% | 1% | 1% |
| QALYs | -719 (-7133 ; 3897)* | -0.081 (-0.15 ; -0.017)* | 8867 | 0% | 0% | 61% | 39% |
| **Including outlier (SA2)** |  |  |  |  |  |  |  |
| Body weight | -2993 (-10852 ; 2800)* | -3.66 (-6.18 ; -1.14)***^,^** ^†^ | 818 | 18% | 82% | 0% | 0% |
| QALYs | -2993 (-10852 ; 2800)* | -0.033 (-0.079 ; 0.012) ***^,^**^†^ | 89376 | 1% | 6% | 76% | 17% |
| CI = confidence interval, , ICER = incremental cost-effectiveness ratio, NE = north-east quadrant, NW = north-west quadrant, SA1 = sensitivity analysis 1, SA2 = sensitivity analysis 2, SE = south-east quadrant, SW = south-west quadrant, QALY = quality adjusted life-year  *Difference in costs and effects is adjusted for clustering of FACT team. The difference in weight is additionally adjusted for repeated observations.  ^†^Covariates included in the mixed model for weight were treatment indicator, baseline weight, gender, diagnosis of mental disorder, having a partner and smoking. The mixed model for QALYs was adjusted for baseline utility. | | | | | | | |

Figure 1. Cost-effectiveness planes and cost-effectiveness acceptability curves

# Discussion

Overall, the SMILE intervention resulted in lower costs over the 12-month follow-up period compared to the usual care group, but not in more QALYs gained. For body weight change the SMILE-intervention was dominant over usual care with the probability of the intervention being cost-effective compared to usual care being 92% at a ceiling ratio of 1,000 euro per additional kilogram body weight loss.

The SMILE intervention was not more effective in improving quality of life. Quality of life is a frequently reported patient-reported outcome in lifestyle interventions in this target population. However, a systematic review showed that most studies did not report a clinically relevant effect on this outcome [15]. Two studies included in this systematic review described large effects. The lifestyle interventions in these studies consisted of high intensity exercise activities that included social interactions, such as soccer practice. The SMILE intervention included low intensity activities (primarily walking) and social activities, as it was conducted in groups. Although these were important aspects of the intervention, there was less focus on social activities and the intensity of exercise was lower compared to the two studies in the systematic review that showed large effects on quality of life [15].

Evidence shows that the EQ-5D has adequate item functioning, reliability, validity and responsiveness for patients with mild, moderate and major depression [47, 48]. However, studies are inconclusive about the use of the EQ-5D questionnaire for people with schizophrenia, which is the most common diagnosis within the SMI population [48, 49]. Using the EQ-5D in SMI populations has been shown to be challenging [50]. Research has indicated that available generic quality of life measures may not be appropriate for people with mental health problems [51, 52]. Future studies could consider the use of bolt-on dimensions when working with the EQ-5D by adding additional dimensions relevant to the SMI population to the existing five dimensions of the measure (mobility, self-care, usual activities, pain/discomfort and anxiety/depression). Using bolt-on dimensions makes the questionnaire more responsive for other dimensions, such as psycho-social well-being, vitality, and relationship dimensions that are not included in the EQ-5D, and possibly make a better ‘fit’ for people with SMI [53, 54].

Interestingly, for the SMILE intervention we found lower costs for home care (-€1724 (95%CI -6519 ; 504) and higher costs for informal care €611 (95%CI 165 ; 1117) in comparison to the usual care group. Patients might have become more independent due to the intervention or more empowered to ask for and receive more informal care. Consequently, this may have led to a reduction of the need for homecare. However, we did not expect such a large difference in home care and informal care costs and cannot rule out the possibility that these differences (partly) occurred by chance.

## Strengths and limitations

Our study was performed with a pragmatic design in a real world setting with the same available resources and time as usual practice and, therefore, contributing to the generalizability of results. In addition, a societal perspective was used in this study. This entails that all costs were collected regardless of who had paid for them. This provides the opportunity to identify shifting of costs between sectors (e.g., healthcare and social affairs and employment). Furthermore, we performed multiple imputation and used a multi-level regression analysis to account for clustering in the data, which is recommended in literature but not commonly performed [40, 55].

There are also some limitations of this study. We found an outlier that strongly influenced the results. One patient in the control group received 20 hours of home care for a consecutive 24 weeks each day. With this outlier included, cost saving increased from €719 to €2993, which consequently changed the ICERs. We asked healthcare professionals what could have been the reason for this high amount of home care, and they considered it to be unlikely. We therefore excluded this patient from the main analysis.

## Implications for future research, practice and policy makers

Knowledge about the cost-effectiveness of lifestyle interventions for people with SMI is essential for informing policy makers. Currently, literature on cost-effectiveness analysis of lifestyle interventions for people with SMI is lacking [13, 21, 22]. Therefore, this study offers an important contribution to the scarce literature available.

It is known that people with SMI may need more support in comparison to the general population to overcome barriers associated with their health conditions such as cognitive deficits [56] and lower health literacy [57], which presumably translates into more investment and thus higher costs. It is promising that we found lower costs for the SMILE intervention compared to usual care, making lifestyle interventions targeting people with SMI more of interest. In the Netherlands, where FACT-teams are an important healthcare setting for people with SMI, implementation of the SMILE lifestyle intervention might be opportune if weight loss is considered the most important outcome, especially considering the results of our process evaluation and qualitative study. The intervention was well perceived by clients and mental healthcare professionals who delivered the intervention. They also considered the SMILE intervention feasible [24, 25]. However, it does not seem opportune to implement the SMILE intervention if QALYs are considered more important. More cost-effectiveness studies in other countries and other settings are needed to gain further insight into the cost-effectiveness of lifestyle interventions for people with SMI.

# Conclusion

The SMILE intervention was less costly compared to usual care and cost-effective for body weight change. However, the SMILE intervention does not seem to be cost-effective when QALYs are the main outcome.

# References

1.	Nielsen RE, Banner J, Jensen SE. Cardiovascular disease in patients with severe mental illness. Nature Reviews Cardiology. 2021;18(2):136-45.

2.	Nordentoft M, Wahlbeck K, Hällgren J, Westman J, Ösby U, Alinaghizadeh H, et al. Excess mortality, causes of death and life expectancy in 270,770 patients with recent onset of mental disorders in Denmark, Finland and Sweden. PloS one. 2013;8(1):e55176.

3.	Lomholt LH, Andersen DV, Sejrsgaard-Jacobsen C, Øzdemir CM, Graff C, Schjerning O, et al. Mortality rate trends in patients diagnosed with schizophrenia or bipolar disorder: a nationwide study with 20 years of follow-up. International Journal of Bipolar Disorders. 2019;7(1):1-8.

4.	Hayes J, Miles J, Walters K, King M, Osborn D. A systematic review and meta‐analysis of premature mortality in bipolar affective disorder. Acta Psychiatrica Scandinavica. 2015;131(6):417-25.

5.	Laursen TM, Nordentoft M, Mortensen PB. Excess early mortality in schizophrenia. Annual review of clinical psychology. 2014;10:425-48.

6.	Walker ER, McGee RE, Druss BG. Mortality in mental disorders and global disease burden implications: a systematic review and meta-analysis. JAMA psychiatry. 2015;72(4):334-41.

7.	Nielsen RE, Uggerby AS, Jensen SOW, McGrath JJ. Increasing mortality gap for patients diagnosed with schizophrenia over the last three decades—a Danish nationwide study from 1980 to 2010. Schizophrenia research. 2013;146(1-3):22-7.

8.	Westman J, Hällgren J, Wahlbeck K, Erlinge D, Alfredsson L, Ösby U. Cardiovascular mortality in bipolar disorder: a population-based cohort study in Sweden. BMJ open. 2013;3(4):e002373.

9.	Jayatilleke N, Hayes RD, Dutta R, Shetty H, Hotopf M, Chang C-K, et al. Contributions of specific causes of death to lost life expectancy in severe mental illness. European psychiatry. 2017;43:109-15.

10.	Miller MJ, Ambrose DM. The Problem of Missed Mental Healthcare Appointments. Clinical schizophrenia & related psychoses. 2019;12(4):177-84.

11.	Vancampfort D, Firth J, Schuch FB, Rosenbaum S, Mugisha J, Hallgren M, et al. Sedentary behavior and physical activity levels in people with schizophrenia, bipolar disorder and major depressive disorder: a global systematic review and meta-analysis. World psychiatry : official journal of the World Psychiatric Association (WPA). 2017;16(3):308-15.

12.	Vázquez-Bourgon J, Pérez-Iglesias R, Ortiz-García de la Foz V, Suárez Pinilla P, Díaz Martínez Á, Crespo-Facorro B. Long-term metabolic effects of aripiprazole, ziprasidone and quetiapine: a pragmatic clinical trial in drug-naïve patients with a first-episode of non-affective psychosis. Psychopharmacology. 2018;235(1):245-55.

13.	Firth J, Siddiqi N, Koyanagi A, Siskind D, Rosenbaum S, Galletly C, et al. The Lancet Psychiatry Commission: a blueprint for protecting physical health in people with mental illness. The Lancet Psychiatry. 2019;6(8):675-712.

14.	Nugter MA, Engelsbel F, Bähler M, Keet R, van Veldhuizen R. Outcomes of FLEXIBLE assertive community treatment (FACT) implementation: a prospective real life study. Community mental health journal. 2016;52(8):898-907.

15.	Pape LM, Adriaanse MC, Kol J, van Straten A, van Meijel B. Patient-reported outcomes of lifestyle interventions in patients with severe mental illness: a systematic review and meta-analysis. BMC Psychiatry. 2022;22(1):261.

16.	Firth J, Solmi M, Wootton RE, Vancampfort D, Schuch FB, Hoare E, et al. A meta-review of "lifestyle psychiatry": the role of exercise, smoking, diet and sleep in the prevention and treatment of mental disorders. World psychiatry : official journal of the World Psychiatric Association (WPA). 2020;19(3):360-80.

17.	Naslund JA, Whiteman KL, McHugo GJ, Aschbrenner KA, Marsch LA, Bartels SJ. Lifestyle interventions for weight loss among overweight and obese adults with serious mental illness: a systematic review and meta-analysis. General hospital psychiatry. 2017;47:83-102.

18.	Jakobsen AS, Speyer H, Nørgaard HCB, Karlsen M, Birk M, Hjorthøj C, et al. Effect of lifestyle coaching versus care coordination versus treatment as usual in people with severe mental illness and overweight: Two-years follow-up of the randomized CHANGE trial. PLoS One. 2017;12(10):e0185881.

19.	Deenik J, van Lieshout C, van Driel HF, Frederix GWJ, Hendriksen IJM, van Harten PN, et al. Cost-Effectiveness of a Multidisciplinary Lifestyle-Enhancing Treatment for Inpatients With Severe Mental Illness: The MULTI Study V. Schizophrenia Bulletin Open. 2022;3(1).

20.	Bartels SJ. Can behavioral health organizations change health behaviors? The STRIDE study and lifestyle interventions for obesity in serious mental illness. Am Psychiatric Assoc; 2015.

21.	Stubbs B, Vancampfort D, Hallgren M, Firth J, Veronese N, Solmi M, et al. EPA guidance on physical activity as a treatment for severe mental illness: a meta-review of the evidence and Position Statement from the European Psychiatric Association (EPA), supported by the International Organization of Physical Therapists in Mental Health (IOPTMH). European Psychiatry. 2018;54:124-44.

22.	Czosnek L, Lederman O, Cormie P, Zopf E, Stubbs B, Rosenbaum S. Health benefits, safety and cost of physical activity interventions for mental health conditions: A meta-review to inform translation efforts. Mental Health and Physical Activity. 2019;16:140-51.

23.	Walburg FS, van Meijel B, van Tulder MW, Adriaanse MC. Cost-effectiveness of a lifestyle intervention for people with a serious mental illness (SMILE): design of a pragmatic cluster-randomised controlled trial. BMC Psychiatry. 2019;19(1):151.

24.	Walburg FS, de Joode JW, Brandt HE, van Tulder MW, Adriaanse MC, van Meijel B. Implementation of a lifestyle intervention for people with a severe mental illness (SMILE): a process evaluation. BMC health services research. 2022;22(1):1-12.

25.	Walburg F, de Joode JW, Brandt H, van Tulder M, Adriaanse M, van Meijel B. Experiences and perceptions of people with a severe mental illness and health care professionals of a one-year group-based lifestyle programme (SMILE). PloS one. 2022;17(8):e0271990.

26.	Walburg FS, van Meijel B, Hoekstra T, Kol J, Pape LM, de Joode JW, et al. Effectiveness of a Lifestyle Intervention for People With a Severe Mental Illness in Dutch Outpatient Mental Health Care: A Randomized Clinical Trial. JAMA Psychiatry. 2023;80(9):886-94.

27.	Yarborough BJH, Leo MC, Stumbo S, Perrin NA, Green CA. STRIDE: a randomized trial of a lifestyle intervention to promote weight loss among individuals taking antipsychotic medications. BMC psychiatry. 2013;13(1):238.

28.	Yarborough BJH, Leo MC, Yarborough MT, Stumbo S, Janoff SL, Perrin NA, et al. Improvement in body image, perceived health, and health-related self-efficacy among people with serious mental illness: the STRIDE study. Psychiatric Services. 2016;67(3):296-301.

29.	Green CA, Yarborough BJH, Leo MC, Yarborough MT, Stumbo SP, Janoff SL, et al. The STRIDE weight loss and lifestyle intervention for individuals taking antipsychotic medications: a randomized trial. The American journal of psychiatry. 2015;172(1):71-81.

30.	Feng Y-S, Kohlmann T, Janssen MF, Buchholz I. Psychometric properties of the EQ-5D-5L: a systematic review of the literature. Quality of Life Research. 2021;30(3):647-73.

31.	Versteegh MM, Vermeulen KM, Evers SM, De Wit GA, Prenger R, Stolk EA. Dutch tariff for the five-level version of EQ-5D. Value in health. 2016;19(4):343-52.

32.	Herdman M, Gudex C, Lloyd A, Janssen M, Kind P, Parkin D, et al. Development and preliminary testing of the new five-level version of EQ-5D (EQ-5D-5L). Quality of life research. 2011;20(10):1727-36.

33.	(CBS) SN. Dutch consumer prices: Statistics Netherlands (CBS); 2020 [Available from: <https://www.cbs.nl/en-gb/series/consumer-prices>.

34.	Bouwmans C, De Jong K, Timman R, Zijlstra-Vlasveld M, Van der Feltz-Cornelis C, Tan SS, et al. Feasibility, reliability and validity of a questionnaire on healthcare consumption and productivity loss in patients with a psychiatric disorder (TiC-P). BMC health services research. 2013;13(1):1-9.

35.	Hakkaart-van Roijen L, Van der Linden N, Bouwmans C, Kanters T, Tan SS. Kostenhandleiding.

36.	van Buuren S, Groothuis-Oudshoorn K. mice: Multivariate Imputation by Chained Equations in R. 2011. 2011;45(3):67.

37.	Faria R, Gomes M, Epstein D, White IR. A Guide to Handling Missing Data in Cost-Effectiveness Analysis Conducted Within Randomised Controlled Trials. PharmacoEconomics. 2014;32(12):1157-70.

38.	White IR, Royston P, Wood AM. Multiple imputation using chained equations: Issues and guidance for practice. Statistics in Medicine. 2011;30(4):377-99.

39.	Rubin DB. Multiple imputation for nonresponse in surveys. New York ;: Wiley; 1987. xxix, 258 p. p.

40.	El Alili M, van Dongen JM, Goldfeld KS, Heymans MW, van Tulder MW, Bosmans JE. Taking the Analysis of Trial-Based Economic Evaluations to the Next Level: The Importance of Accounting for Clustering. PharmacoEconomics. 2020;38(11):1247-61.

41.	Twisk JW. Applied multilevel analysis: a practical guide for medical researchers: Cambridge University Press; 2006.

42.	Twisk JW. Applied longitudinal data analysis for epidemiology: a practical guide: cambridge university press; 2013.

43.	Manca A, Hawkins N, Sculpher MJ. Estimating mean QALYs in trial‐based cost‐effectiveness analysis: the importance of controlling for baseline utility. Health economics. 2005;14(5):487-96.

44.	Fenwick E, O'Brien BJ, Briggs A. Cost-effectiveness acceptability curves--facts, fallacies and frequently asked questions. Health Econ. 2004;13(5):405-15.

45.	Hoch JS, Dewa CS. Advantages of the net benefit regression framework for economic evaluations of interventions in the workplace: a case study of the cost-effectiveness of a collaborative mental health care program for people receiving short-term disability benefits for psychiatric disorders. Journal of occupational and environmental medicine. 2014;56(4):441-5.

46.	Karpenko AW, Geenen JW, Vreman RA, Hovels A. The Introduction Of A Threshold For The Icer And The Implications For Reimbursement Of Drugs In The Dutch Healthcare System. Value in Health. 2017;20(9):A671.

47.	Bilbao A, Martín-Fernández J, García-Pérez L, Mendezona JI, Arrasate M, Candela R, et al. Psychometric properties of the EQ-5D-5L in patients with major depression: factor analysis and Rasch analysis. Journal of Mental Health. 2021:1-11.

48.	Brazier J. Is the EQ–5D fit for purpose in mental health? The British Journal of Psychiatry. 2010;197(5):348-9.

49.	Mulhern B, Mukuria C, Barkham M, Knapp M, Byford S, Brazier J. Using generic preference-based measures in mental health: psychometric validity of the EQ-5D and SF-6D. The British Journal of Psychiatry. 2014;205(3):236-43.

50.	Delespaul PH. Consensus over de definitie van mensen met een ernstige psychische aandoening (EPA) en hun aantal in Nederland. Tijdschrift voor Psychiatrie. 2013;55(6):427-38.

51.	Connell J, O'Cathain A, Brazier J. Measuring quality of life in mental health: are we asking the right questions? Soc Sci Med. 2014;120:12-20.

52.	Connell J, Brazier J, O’Cathain A, Lloyd-Jones M, Paisley S. Quality of life of people with mental health problems: a synthesis of qualitative research. Health and quality of life outcomes. 2012;10(1):1-16.

53.	Finch AP, Brazier JE, Mukuria C. Selecting bolt-on dimensions for the EQ-5D: examining their contribution to health-related quality of life. Value in Health. 2019;22(1):50-61.

54.	Chen G, Olsen JA. Filling the psycho-social gap in the EQ-5D: the empirical support for four bolt-on dimensions. Quality of Life Research. 2020;29(11):3119-29.

55.	Gomes M, Grieve R, Nixon R, Edmunds W. Statistical methods for cost-effectiveness analyses that use data from cluster randomized trials: a systematic review and checklist for critical appraisal. Medical Decision Making. 2012;32(1):209-20.

56.	Fioravanti M, Bianchi V, Cinti ME. Cognitive deficits in schizophrenia: an updated metanalysis of the scientific evidence. BMC psychiatry. 2012;12(1):1-20.

57.	Clausen W, Watanabe-Galloway S, Bill Baerentzen M, Britigan DH. Health literacy among people with serious mental illness. Community mental health journal. 2016;52(4):399-405.
